# Cost-of-illness studies in heart failure: a systematic review 2004–2016

**DOI:** 10.1186/s12872-018-0815-3

**Published:** 2018-05-02

**Authors:** Wladimir Lesyuk, Christine Kriza, Peter Kolominsky-Rabas

**Affiliations:** 1 Centre for Health Technology Assessment (HTA) and Public Health (IZPH), Friedrich-Alexander-University Erlangen-Nürnberg, Erlangen, Germany; 2 National Leading-Edge Cluster Medical Technologies ‘Medical Valley EMN’, Erlangen, Bavaria Germany

**Keywords:** Heart failure, Cost-of-illness, Economic burden, Heart disease

## Abstract

**Background:**

Heart failure is a major and growing medical and economic problem worldwide as 1–2% of the healthcare budget are spent for heart failure. The prevalence of heart failure has increased over the past decades and it is expected that there will be further raise due to the higher proportion of elderly in the western societies. In this context cost-of-illness studies can significantly contribute to a better understanding of the drivers and problems which lead to the increasing costs in heart failure.

The aim of this study was to perform a systematic review of published cost-of-illness studies related to heart failure to highlight the increasing cost impact of heart failure.

**Methods:**

A systematic review was conducted from 2004 to 2016 to identify cost-of-illness studies related to heart failure, searching PubMed (Medline), Cochrane, Science Direct (Embase), Scopus and CRD York Database.

**Results:**

Of the total of 16 studies identified, 11 studies reported prevalence-based estimates, 2 studies focused on incidence-based data and 3 articles presented both types of cost data. A large variation concerning cost components and estimates can be noted. Only three studies estimated indirect costs. Most of the included studies have shown that the costs for hospital admission are the most expensive cost element. Estimates for annual prevalence-based costs for heart failure patients range from $868 for South Korea to $25,532 for Germany. The lifetime costs for heart failure patients have been estimated to $126.819 per patient.

**Conclusions:**

Our review highlights the considerable and growing economic burden of heart failure on the health care systems. The cost-of-illness studies included in this review show large variations in methodology used and the cost results vary consequently. High quality data from cost-of-illness studies with a robust methodology applied can inform policy makers about the major cost drivers of heart failure and can be used as the basis of further economic evaluations.

**Electronic supplementary material:**

The online version of this article (10.1186/s12872-018-0815-3) contains supplementary material, which is available to authorized users.

## Background

### The burden of heart failure

Heart failure (HF) is a major and growing medical and economic problem, with high prevalence and incidence rates worldwide [1, 2]. HF is defined as a pathophysiological state in which an abnormality of cardiac function is responsible for the failure of the heart to pump blood at a rate commensurate with the requirements of the metabolizing tissues [3]. It has been estimated that 0.4–2.2% of the population in industrialized countries suffer from HF [4], with between 500,000–600,000 incident cases diagnosed each year [5]. HF affects especially the elderly, with 80% of HF-related hospitalizations and 90% of HF-related deaths occurring among patients aged 65 years or older [6]. The prevalence of HF has increased over the past decades [7]. It is expected that there will be a further rise due to a higher proportion of elderly people and better survival rates of patients with conditions such as hypertension, diabetes, etc., which trigger the development of HF [7, 8]. In addition, two thirds of the patients are readmitted to hospital within one year [9]. The mortality rate for patients with HF is high, as showed in the MAGGIC meta-analysis includes individual data on 39,372 patients with 40,2% died during a median follow-up of 2,5 years [10]. A recently published study demonstrated that the 30-day readmission rates for HF are higher than for pneumonia or acute myocardial infarction [11].

Due to the high and increasing prevalence rates, HF constitutes an enormous economic burden for the healthcare systems in industrialized countries. For example, Europa and USA spent 1–2% of their annual healthcare budget on HF [6]. The global economic burden of HF is estimated at $108 billons per annum, with $65 billons attributed to direct and $43 billons to indirect costs [12]. The US is the biggest contributor to the global HF costs and is responsible for 28.4% of total global HF spend [12]. Europa accounts for 6.83% of total global HF costs [12].

Due to the considerable cost impact of HF on healthcare systems, it is necessary to have a better understanding of the cost aspects and the specific cost drivers. In this context, Cost-of-Illness (COI) studies are an important tool to analyze the economic burden of HF and to provide information on the cost drivers to clinicians and health policy makers. On the basis of transparent and detailed cost components this is aimed at improving the planning and development of healthcare services and optimization of the allocation of healthcare expenditures and medical resources [13]. COI studies are often restricted to a certain country, deal with small patient groups or present only a part of all illness costs. Therefore, it is important to summarize the results of different COI-studies in a systematic way.

### Background on cost-of-illness studies

COI studies estimate the resources consumed and lost as a result of a particular disease. Results from the COI studies can improve understanding of the economic burden that a specific disease may have on society as whole, healthcare providers, and the individual patient. COI studies can also provide a fundamental basis for further economic evaluations, such as cost-effectiveness-, cost-utility- and cost-benefit analysis [14].

#### Perspective

COI studies can be conducted from various perspectives. Based on the chosen perspective, the cost estimation can vary. The most popular perspectives are the societal perspective and the viewpoints of the patient, the insurance company or the healthcare providers.

#### Epidemiological approach

COI studies follow two different epidemiological approaches, either the prevalence- or the incidence-based approach. Prevalence-based studies measure costs, which occur with prevalent cases over a specified time period, usually 1 year [15].

The incidence-based approach focusses on lifetime costs attributed to a disease. The costs are measured from the onset of a disease [15].

#### Method of resource quantification

COI studies use two different methods to estimate costs. The bottom-up approach (“person-based”) assigns costs to individuals with the health condition of interest, for example by using data from real cases [15]. The top-down method (“population-based”) allocates parts of aggregated costs to specific diseases.

### Objectives

The aim of this study was to perform a systematic review of recently published COI studies related to HF to highlight the increasing cost impact associated with this disease and identify the major cost drivers.

## Methods

We conducted a systematic literature search for journal articles between 2004 and 2016 in the following databases: PubMed (Medline), Cochrane, Science Direct (Embase), Scopus and CRD (Centre for Reviews and Dissemination) York Database incl. National Health Service Economics Evaluation Database (NHS EED). To identify relevant COI-studies for HF, appropriate disease-related MeSH terms were used (Additional file 1). The references or citations of the retrieved articles were reviewed for additional articles (citation snowballing). The search methodology was in line with the PRISMA (Preferred Reporting Items for Systematic Reviews and Meta-Analyses) guidelines [16] except for the use of the PICOS (population, intervention, comparators, outcomes, study design) review system.

### Inclusion and exclusion criteria

Search results were transferred to EndNote, version X7, and reviewed independently by two researchers. The inclusion and exclusion criteria were adopted from the CHEC list [17] and the BMJ guidelines for authors and peer reviewers of economic submissions [18]. Although the two checklists were developed for the assessment of economic evaluations, we derived criteria that are also relevant for the evaluation of cost-of-illness studies. These criteria are listed in the appendix (Additional file 2). Furthermore they were in accordance with the check list for COI evaluation in the guide to critical evaluation of COI studies developed by Larg et al. [15].

HF can be categorized in two entities, systolic HF and diastolic HF. Systolic heart failure is defined as the entity in which the ejection fraction is reduced. In diastolic HF the ejection fraction is preserved. Owan et al. has shown that 53% of patients suffer from systolic and 47% from diastolic heart failure [19]. As our aim was to analyze all entities of HF independently of the pathophysiologic mechanism, we excluded papers dealing only with systolic/diastolic HF.

### General characteristics of the studies

To provide a comprehensive understanding, the included studies were analyzed in terms of country, epidemiological approach (prevalent vs. incident), study period, perspective (societal, healthcare provider, etc.), main data sources and the identification of HF patients. When the study perspective or the epidemiological approach were not clearly specified in the studies, two investigators achieved consensus by discussion.

### Standardization of costs

The reported costs in the included studies were transferred from the local currency in the year of the costs to the inflated values in local currency for the year 2016 [20]. This cost data was exchanged to US-dollars by using the gross domestic product purchasing power parity (PPP) [21]. This methodology can be used for COI studies in order to reach better comparability between the different currencies [22].

## Results

**Fig. 1:**
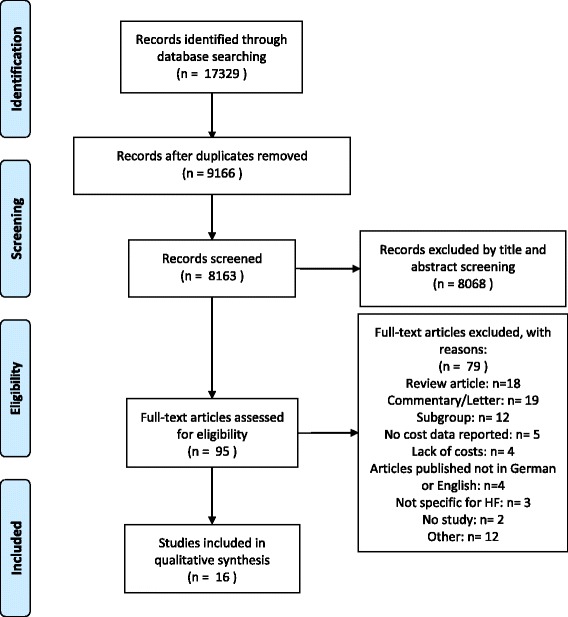
Data acquisition flowchart

The search procedure is shown in the PRISMA Flow Diagram (Fig. 1: Data acquisition flowchart). The systematic literature search identified 17,329 potential relevant articles. After removing 9166 duplicates, there were 8163 studies, which were screened by title and abstract. 8068 papers were excluded because they did not deal with COI studies of HF. Of the remaining 95 articles, another 79 studies did not fulfill the inclusion criteria shown in the Appendix. In all, 16 articles were identified in this review and analyzed by their study characteristics and cost data.

### Study characteristics

Study characteristics are summarized in Tables 1 and 2. Reviewed COI studies showed results for ten countries. Eight studies were conducted in Europa, and six in North America. The study population size ranged from a minimum of 115 to a maximum of 475,019. The mean age of the study population varied from 58 to 81.6 years. Three studies [23–25] reported the mean age of different subgroups (HF-group vs. no-HF control group). Eleven articles had a prevalent and two an incident epidemiological approach. Three studies provided both types of results [25–27]. Twelve studies adopted the perspective of a third-party payer and six articles were categorized as a prospective study.

**Table 1:** COI-studies in HF: Summary of main study characteristics

Reference	Country	Study size	Epidemiological approach	Method of Resource Quantification	Study period	Perspective	Study design	Mean age
Voigt 2014 [35]	USA	–	Prevalent^a^	Mixed^a^	2007–2012	S^a^	R	–
Corrao 2014 [33]	Italy	26,949	Incident	Top-down^a^	2011	P	R	79
Czech 2013 [36]	Poland	–	Prevalent^a^	Mixed^a^	2009–2011	P	R	–
Delgado 2013 [37]	Spain	374	Prevalent^a^	Bottom-up^a^	2010	S	Pr	62
Dunlay 2011 [34]	USA	1054	Incident^a^	Top-down^a^	1987–2006	P^a^	R	76,8
Bogner 2010 [24]	USA	7996	Prevalent^a^	Bottom-up^a^	2000–2001	P	R	77,8–81,4
Zugck 2010 [30]	Germany	86,493	Prevalent^a^	Top-down^a^	2002	P^a^	R	–
Neumann 2009 [29]	Germany	–	Prevalent^a^	Top-down^a^	2000–2007	P^a^	R	–
Liao 2007 [23]	USA	4860	Prevalent	Top-down	1992–2003	P^a^	Pr	75,6- 78,2
Liao 2006 [25]	USA	881	Mixed	Top-down	1992–1998	P^a^	Pr	77,6- 81,6
Agvall 2005 [28]	Sweden	115	Prevalent^a^	Bottom-up^a^	1999–2000	P^a^	R	77
Ory 2005 [26]	USA	17,835	Mixed	Bottom-up^a^	1999–2001	P^a^	Pr	76,4
Stafylas 2016 [38]	Greece	307	Prevalent	Top-down^a^	2009–2011	P	Pr	66
Lee 2016 [31]	South Korea	475,019	Prevalent	Top-down	2014	P / S	R	–
Murphy 2016 [27]	Ireland	1292	Mixed^a^	Mixed^a^	2013	P^a^	R	74,5
Ogah 2014 [32]	Nigeria	239	Prevalent	Mixed^a^	2009–2010	S	Pr	58

^a^not clearly stated in the study, consensus by discussion

S – societal; P – third-party payer; R – retrospective; Pr - prospective

**Table 2:** Main data sources and definition of HF in the included studies

Study	Main data sources	Definition of HF
Voigt, 2014 [35]	Agency for Healthcare Research and Quality (AHRQ) National Association for Home Care & Hospice (NAHC) National Ambulatory Medical Care Survey (NAMCS) National Hospital Ambulatory Medical Care Survey (NHAMCS) Centers for Medicare & Medicaid Services (CMS)	ICD-9 (428.x, 402.01, 402.11, 402.91, 398.91, 404.01, 404.11, 404.91, 416.9, 425.4, 518.4, 786)
Corrao, 2014 [33]	Italian National Health System (NHS) database from Lombardy	ICD-9 (428, 402.01, 402.11, 402.91)
Czech, 2013 [36]	Medical data from randomly selected outpatient units and inpatient facilities linked with patient interview data (POLKARD study)	–
Delgado, 2013	Medical records from specialized cardiology clinics, questionnaires and interviews (patients and caregivers)	Symptomatic patients (NYHA II-IV) with a diagnosis of HF at least 6 months previously
Dunlay, 2011 [34]	Medical records and billing data from Olmsted County Healthcare Expenditure and Utilization Database (OCHEUD), a population-based database in Olmsted County, Minnesota, USA	ICD-9 (428)
Bogner, 2010 [24]	Administrative database of a large urban academic health care system Medicare claims database	ICD-9 (428.0, 428.1, 428.9, 402.01, 402.11, 402.91)
Zugck, 2010 [30]	Database of the public health insurance, cohort selected by randomly prescribed date of birth Federal Office of Statistics, Germany	ICD-10 (I50)
Neumann, 2009 [29]	Federal Office of Statistics, Germany	ICD-10 (I50)
Liao, 2007 [23] Liao, 2006 [25]	Cardiovascular Health Study (prospective, community-based, observational study) Medicare linked files	Hospitalization for HF or self-report of a physician diagnosis of HF
Agvall, 2005 [28]	Hospital records from two healthcare centers Swedish National Medical Agency price list	ICD-10 (I50)
Ory, 2005 [26]	Longitudinal database of Prescription Solutions, a pharmacy benefit and medical management organization	ICD-9 (398.91, 402.01, 402.11, 402.91, 404.01, 404.03, 404.11, 404.91)
Stafylas, 2016	EURObservational Research Programme: The Heart Failure Pilot Survey (ESC-HF Pilot) EOPYY- Greek National Organization for Health Care Provision	Hospitalization for HF or HF diagnosis according to clinical judgement of the responsible cardiologist
Lee, 2016 [31]	Claims data from the National Health Insurance (NHI) Claims data from Medical Aid (MA)	ICD-10 (I11.0, I13.0, I13.2, I50.x)
Murphy, 2016	National Casemix Program, patient interviews, hospital records	ICD-10
Ogah, 2014 [32]	Abeokuta HF registry (hospital registry), patient interviews	ICD-10

To identify HF patients, the studies used different methods. Six articles referred to the ICD-10 (International Classification of Diseases), [27–32], five to the ICD-9 [24, 26, 33–35] and five [23, 25, 36–38] didn’t specify the ICD classification.

Because HF is usually accompanied by many underlying diseases, it is often difficult to identify the correct cases of HF based on the diagnosis codes of ICD. Lee et al. [31] showed that by defining HF only as the primary diagnosis the HF cases decrease by 46% in contrast to define HF cases as primary and secondary diagnosis (using the same ICD-codes). By further analyzes he showed, that 75% of patients with HF, which was defined as a secondary diagnosis had a primary diagnosis which was related to HF, such as hypertension or angina pectoris. Thus, defining HF only as the primary diagnosis might lead to an under-specification of HF [31].

A second study of Voigt et al. [35] calculated costs for HF as the primary diagnosis (HF in isolation, HFI) and HF as one of multiple diagnosis/part of a disease milieu (HF syndrome, HFS). As a consequence, costs range between 70.8 (HFI)- 127.0 (HFS) billion dollars. This might show the great underestimation of economic burden, if defining HF only as the primary diagnosis [35].

### Epidemiological data

HF shows high prevalence rates with 12.4 per 1000 persons suffering from this chronic condition [31]. The prevalence rates increase with age [29, 31, 36], with patients aged 65 or older showed 9.2-fold higher prevalence and 1.6 higher costs than 19–64 aged population [31]. Neumann et al. [29] analyzed that patients 65 or older shows 10-times higher prevalence rates than 45–65 aged patients.

Incidence rates are also high with 2.4–3.8 per 1000 persons [33]. HF results in a severe mortality rate with 1-year mortality after HF hospitalization of 24% [33]. Stafylas et al. [38] showed that the mortality rates are higher for patients, who were hospitalized for HF (24,3%) than for HF patients treated outpatient (7,7%).

The included studies reported high, similar readmission rates for HF between 42% and 44,9% [26, 28, 38].

Of the included articles, two separated the cost data by gender [28, 29] and both reported higher costs for women.

### Cost components

To enable better comparability, prevalence-based studies were grouped separately from the incidence-based ones.

#### Cost components of the prevalence-based approach

The cost components of the included studies were subject to large variations (Table 3). Most of the articles considered the economic burden of HF only in terms of direct costs, such as costs attributed to hospitalization, medication, home care, etc. Two studies [31, 37] additionally accounted for the costs of informal care. Informal caregiving was defined as care provided by individuals who were not professional social or health care workers [37]. The indirect costs in terms of loss of productivity were considered only by three studies [31, 32, 35]. Lee et al. [31] calculated caregiver’s costs as the product of the average annual inpatient days per patient due to HF and the average market price for the daily charge of a helper. He also calculated indirect costs in terms of productivity loss due to morbidity and mortality for ages under 65 by using mathematical equations derived from the human capital approach.

**Table 3:** Summary of the cost components (studies with an incident and mixed approach are underlined)

Cost components	(37)	(29)	(36)	(35)	(28)	(23)	(24)	(30)	(38)	(31)	(32)	(27)	(34)	(33)	(26)	(25)
Direct costs	✓	✓	✓	✓	✓	✓	✓	✓	✓	✓	✓	✓	✓	✓	✓	✓
Inpatient care	✓	✓	✓	✓	✓	✓	✓	✓	✓	✓	✓	✓	✓	✓		✓
Medication				✓			✓	✓	✓	✓	✓	✓	✓			
Laboratory									✓		✓		✓			
Physicians				✓					✓							
Intensive care units			✓		✓											
Nursing home	✓	✓		✓	✓											
Outpatient care	✓	✓	✓	✓	✓	✓	✓	✓	✓	✓	✓	✓	✓	✓		✓
Hospital Outpatient care				✓	✓						✓					
Physicians	✓	✓	✓	✓	✓				✓			✓	✓			
Specialist	✓															
Home care	✓			✓												
Medication	✓	✓	✓	✓	✓			✓	✓	✓	✓			✓	✓	
Laboratory /Procedures					✓				✓		✓		✓	✓		
Paramedical staff		✓		✓	✓											
Medical transport	✓	✓								✓	✓					
Indirect costs				✓						✓	✓					
Informal care costs	✓									✓						

In the prospective, observational study Delgado et al. [37] estimated the costs of informal care by recording the hours of caregiving provided. The maximum number of caregiving hours per person was limited to 112 (16 h per day for 7 days of caregiving weekly). The number of hours was multiplied with the mean costs of an hour of home care.

Ogah et al. [32] calculated the costs for productivity loss by recording the days of lost work and multiplying them with the minimum wage.

Among the aspects of direct costs, hospital admission and medication were examined in most of the papers. Agvall et al. [28] and Czech et al. [36] estimated the costs for treatment in intensive care units apart from the hospitalization costs. There were also differences in the outpatient care cost components. Whereas most of the included studies reported costs for outpatient medication and physician visits, five articles reported cost data for laboratory and procedures [28, 29, 32–34, 38] such as speech and physical therapy. The economic burden of home care was estimated by Voigt and Delgado et al. [35, 37]. Home care referred to care which was provided by professional caregivers at home.

#### Cost components of the incidence-based group

In comparison to the prevalence-based group, the articles with an incidence-based or mixed approach have less cost components (Table 3). All studies included costs for inpatient care, and all except one [25] considered the costs for medication. None of the studies estimated the costs for home care, nursing homes, informal caregiving or indirect costs.

### Cost estimates

The main economic estimates are presented in Table 4. The total annual costs per patient ranged from $868 for South Korea [31] to $25,532 for Germany [30]. Two articles [29, 35] accounted the costs not per patient, but analyzed the whole economic burden of HF per year in an aggregated way. The hospitalization costs are the greatest cost component of overall healthcare costs. Among total healthcare costs, expenditures for medication are the second largest issue [33, 37]. Due to hospitalization costs, room and board were the greatest contributor (43% of inpatient costs), following by procedures, imaging and laboratory testing [34]. Dialysis was responsible for the highest part of procedural costs, but it was needed only by a small number of patients [34].

**Table 4:** Summary of cost estimates (studies with an incident and mixed approach are underlined)

Reference	Year of cost data	Country	Reported annual costs in local currency (costs per patient)	Local currency in 2016	$US (2016 PPP)	% of inpatient costs of all direct costs	Expenditure on health, per capita, US$ (2016 PPP)
Voigt, 2014 [35]	2012	USA	$60.2 - $115.4b^a^(direct costs) $70.8 - $127.0b^a^(total costs)	$62.9 - $120.7b^a^ $74.0 - $133.0b^a^	62.9–120.7b^a^ 74.0–133.0b^a^	66	9892
Czech, 2013 [36]	2010	Poland	7739 PLN	8312 PLN	4755	92^e^	1798
Delgado, 2013	2010	Spain	4860€ (healthcare costs)	5166€	7792	58^e^	3248
Bogner, 2010 [24]	2009	USA	22,230$^b^	24,873$^b^	24,873^b^	84	9892
Zugck, 2010 [30]	2002	Germany	11,794–16,303 €^c^	14,297–19,762 €^c^	18,472–25,532^c^	72	5551
Neumann, 2009 [29]	2006	Germany	2.879b €^a^	3.293b €^a^	4.255b^a^	60	5551
Liao, 2007 [23]	2006	USA	$10,832	12,907$	12,907	65^e^	9892
Agvall, 2005 [28]	2000	Sweden	37,060 SEK	44,971 SEK	5044	47	5488
Stafylas, 2016	2014	Greece	4411 €	4295 €	7053	73^e^	2223
Ogah, 2014 [32]	2010	Nigeria	2128$	2343$	2343	44	NA
Lee, 2016 [31]	2016	South Korea	868$ (perspective of third-party-payer) 1414$ (perspective of society)	868$ 1414$	868 1414	53	NA
Dunlay, 2011 [34]	2007	USA	109.541$ (lifetime costs from HF diagnosis until death)	126.819$	126.819	77	9892
Corrao, 2014 [33]	2011	Italy	11,100 €	11,597 €	15,952	92^e^	3391
Liao, 2006 [25]	2000	USA	32,580–33,023$ (prevalent group)^d^ 45,604–49,128$ (incident group)^d^	45,406–46,023$^d^ 63,557–68,468$^d^	45,406–46,023^d^ 63,557–68,468^d^	65–67 70–72	9892
Ory, 2005 [26]	2000	USA	14,465$ (prevalent group) 17,744$ (incident group)	20,159$ 24,729$	20,159 24,729	NA	9892
Murphy, 2016	2013	Ireland	12,206 € (patients with preserved EF) 13,011 € (patients with reduced EF)	12,194 € 12,999 €	15,334 16,330	92^e^ 96^e^	5528

^a^Aggregated costs for all HF patients

^b^Costs aggregated for two years

^c^Costs depend on number of visits to doctors

^d^Cumulated costs for 5 years

^e^not clearly stated in the study

*SEK* Swedish kronas, *PLN* Polish Zloty, *b* Billions, *EF* ejection fraction

The study of Delgado et al. [37] considered the burden of costs of informal caregiving per patient. He estimated that 59.1 to 69,8% were due to informal care costs. Additionally, he showed that the healthcare and informal care costs for HF were rapidly increasing with the number of hospital admissions, contributing to overall increases in total costs for HF. Of the included studies Dunlay et al. [34] presented the lifetime costs for HF for the longest study period.

Ory et al. [26] reported that newly diagnosed patients had significantly higher healthcare charges than the prevalent group. Furthermore, he described that – in comparison to the control group without HF- the patients with HF created four times higher total healthcare costs.

The study of Ogah et al. [32] was the only one conducted in a low/middle income country (Nigeria). 46% of the total costs were contributable to inpatient and 54% to outpatient costs [32]. Inpatient costs were lower than in high-income countries because in these countries the utilization of expensive medical equipment and surgery are higher. Ogah et al. [32] showed that 90% of direct outpatient costs are due to medication and transportation costs for monthly follow-up visits, which were mostly made through out-of-pocket payments.

### Predictors of increasing costs

Some studies estimated cost predictors, which are shown in Table 5. A higher NYHA stage [23, 25, 37, 38], kidney dysfunction [25, 38] and the comorbidity of HF and diabetes mellitus [24, 34] were considered as the most common reasons of increasing costs for HF patients. Two studies [23, 33] reported that that comorbid conditions of HF were much more associated with greater costs than prevalent HF alone. Comorbidities in HF cause ¾ of all readmissions in HF patients [33]. Dunlay et al. [34] conducted a study with an incident epidemiological approach and analyzed that diabetes mellitus caused an increase in lifetime costs of HF patients of 25%. Bogner et al. [24] also emphasized that diabetes mellitus has substantial influence on the costs of managing HF patients, extends the hospital stay and worsens the prognosis. A study analyzed that a preserved ejection fraction (> = 50%) in HF patients leads to an increasing cost impact of 24% [34]. In contrast, Liao et al. [25] showed that there was no statistically significant difference between HF patients with normal and reduced EF due to the 5-year cumulative costs.

**Table 5:** Predictors of increasing costs

Reference	Predictors of increasing costs (x times higher costs)
Stafylas, 2016	• NYHA stage • Kidney dysfunction
Lee, 2016 [31]	• Age > = 65 (1.6) • Number of hospitalizations (9.7 for one hospitalization)
Bogner, 2010 [24]	• Diabetes mellitus (0.4)
Dunlay, 2011 [34]	• Diabetes mellitus (0.25) • Ejection fraction > = 50 (0.24)
Liao, 2006 [25]	• NYHA stage (NYHA 4–0.77, NYHA 3–0.12) • Kidney dysfunction (creatinine > = 1.4 mg/dl – 0.48) • Coronary artery disease (0.32) • COPD (0.38) • Hypertension (0.27)
Liao, 2007 [23]	• NYHA stage (NYHA 3/4–0.41) • Coronary artery disease (0.66) • Kidney dysfunction (0.13) • COPD (0.44)
Delgado, 2013	• NYHA stage (NYHA 3/4: 0.6–0.8 times higher costs than NYHA 2)

The severity of HF is classified by NYHA (New York Heart Association) stages [1]. Of the included studies, only two [36, 37] separated the cost data by NYHA stage (Table 6). Czech et al. [36] gave very detailed information on cost data by NYHA class I-IV, estimating the total annual costs as well as the average costs of hospitalization for HF by NYHA stage. Delgado et al. [37] reported economic data for NYHA stages II-IV and estimated the total costs for HF by combining the NYHA groups III and IV. He emphasized the rising costs and the significantly increased use of health services and social services, as formal and informal care, by NYHA stage. Both studies showed that the economic burden of HF is dependent on the NYHA stage and the costs rise with advanced stages, disregarding NYHA stage I. Thus, it is important to act early and to prevent the progression of HF to more advanced and highly symptomatic forms. Czech et al. [36] reported for NYHA I high annual as well as hospitalization costs and assumed that this could be attributable to the higher proportion of special cardiology interventions in this group. Additionally, he described that NYHA IV was responsible for more than 70% of total annual costs for HF.

**Table 6:** Costs by NYHA stage

Reference	Year of cost data	Country	NYHA I	NYHA II	NYHA III	NYHA IV	Total costs per patient and year
			local currency in year of costs/ local currency in 2016/ $US in 2016, PPP				
Delgado, 2013 (direct costs)	2010	Spain	–	3789€/ 4028€/ 6075$	6832€/ 7262€/ 10,953$		4860€/ 5166€/ 7792$
Delgado, 2013 (total costs)	2010	Spain	–	10,283–14,459€/ 10,931–15,370€/ 16,487–23,183$	18,265–23,721€/ 19,416–25,215€/ 29,285–38,032$		12,995–18,220€/ 13,814–19,368€/ 20,836–29,213$
Czech, 2013 [36]	2010	Poland	a	5,315PLN/ 5,708PLN/ 3265$	8,116PLN/ 8,717PLN/ 4987$	21,273PLN/ 22,847PLN/ 13,070$	7739PLN/ 8312PLN/ 4755$

a Costs are reported, but not listed here

*PLN* Polish Zloty

### Distribution of costs

Two articles [25, 34] compared the cost data of patients in the first year of HF diagnosis to the previous year in order to investigate the impact of HF on the costs (Table 7). Dunlay et al. [34] reported an 318% increase in costs in the year of HF diagnosis. He also examined that the costs for HF were high at the time of initial diagnosis, decreased then, reached a stable, relative low level and increased again at the end of life. Liao et al. [25] estimated in his study that the development of HF had a greater than 200% increase in total costs compared to the year before diagnosis. This strong rise was especially generated by the inpatient cost component. After that he detected a decrease of costs, but they were still higher than in the year before HF diagnosis out to year 5, if only survivors were examined.

**Table 7:** Comparison of costs

Reference	Year of costs	Country	Year prior to HF diagnosis	Year beginning with HF diagnosis	Difference in costs (local currency in year of costs)	Difference in costs (local currency in 2016)	Difference in costs ($US in 2016, PPP)	Raise of the costs in %
Dunlay, 2011 [34]	2007	USA	8219$	34,372$	26,153$	30,278$	30,278	318
Liao, 2006 [23]	2000	USA	6650–6752$	24,882–25,503$	18,232–18,751$	25,409–26,133$	25,409–26,133	274–278

## Discussion

Our systematic review highlights the economic impact of HF as a rising burden for high-income countries. Furthermore, it also uncovers the large heterogeneity of COI studies focused on HF.

### Comparison of the cost data

The main findings of our review are reported in Table 4 and are focused on the highest annual per-patient costs reported for the USA [24] and Germany [30]. Our review shows that the costs for hospital admission contribute significantly to the overall direct costs for HF (ranging from 44 to 96%). Previously published studies accounted that about two-thirds of the direct HF healthcare costs are due to hospitalization [6, 39]. The high inpatient costs are a result of high readmission rates, with 23% of HF patients readmitted to hospital stay within 6 months [6]. A further aspect of our analyses is the considerable increase in costs with advanced NYHA stage, with NYHA stage IV being the most expensive. Biermann et al. [5] estimated similar results for patients with systolic HF. An earlier review [40] estimated that patients with NYHA IV produce between 8 and 30 times higher healthcare costs than patients with NYHA II. We have shown that costs rise rapidly after a confirmed HF diagnosis (Table 7). Although HF is a chronic condition causing high lifetime costs, particularly the first year after a HF diagnosis and the end-of-life care are the most expensive ones. Two studies from the USA [41] and Canada [42] analyzed the last 180 days of life of HF patients and concluded that in the last six months of life, there is a large increase in costs and resource use. Unroe et al. [41] as well as Kaul et al. [42] analyzed that the costs during the last 180 days rose from $28,766 to $36,215 between the years 2000–2007 for the USA [41] and from $25,069 to $27,983 (Canadian dollars) between the years 2000–2006 for Canada [42].

Other studies also report a rise in costs relating to HF over time, but especially in the last two decades. Stewart et al. [39] reported a rise of direct medical costs from £716 million in 1995 to £905 million in 2000 for the UK. Liao et al. [6] presented an increase of HF-related costs for different countries (Spain, Canada, Sweden and Scotland) by 40–71%.

### The need for standards for future COI-studies in HF

Our review highlighted not only the large economic burden of HF, but also the heterogeneity of the studies and lack of cost data. For better comparison of research data, future COI studies should use a standardized approach regarding methodology, in particular regarding criteria concerning the selection of HF patients and data, the inclusion of different cost drivers and the presentation of results.

Thus, further COI studies in HF should clearly state the ICD codes, which were used for identification of HF patients. Additionally, an attribution of cost data to the specific ICD code number could achieve a better comparison between COI studies. As presented in recently published papers, using different ICD codes may lead to an over- or underestimation of HF diagnosis [43, 44].

COI studies should present cost data for the whole HF group and disaggregate them consistently for sub-entities, as systolic or diastolic HF. Importantly, COI studies should emphasize clearly stating the use of the exact diagnoses (including relevant ICD codes). It should be clearly stated, if HF is defined as the primary or secondary diagnosis, as this difference influences the cost estimates. The inclusion of comorbidities would give detailed information on the cost drivers and show opportunities for decreasing costs.

In addition, the study perspective and a distinction between COI approaches should be indicated [18]. This review emphasizes that indirect costs are a significant contributor to total costs and more effort is needed to estimate these costs accurately and consistently. In addition, informal care costs are an important contributor to the COI of HF [37].

As there are many differences between healthcare systems in different countries, high-quality cost data is needed from COI studies to facilitate comparisons between countries and cost trends. Crucially, more robust data from future COI studies is needed to provide a sound basis for cost-effectiveness studies to identify the most cost-effective therapies.

Another aspect in our systematic review highlights that most of the studies are conducted in Europe and USA. But as HF is a global problem, we need more COI-studies from low-and middle-income countries as demanded in a recently published study [45].

## Study limitations

The cost data we included in our systematic review derived from a heterogeneous set of studies with different methodologies used, to collect the data. This lack of a standard method for collecting the cost data may impact some of our findings. The included studies were conducted in different countries with various healthcare systems. This may lead to an over- or underestimation of some cost data, as the included studies not clearly describe, which costs are included in their analyses. For example, some studies present the costs in a detailed way, whereas other studies aggregate particular costs to one cost position.

## Conclusion

Our review highlights the considerable and growing economic burden of HF on the health care systems of industrialized countries. The trend for rising costs has especially been more significant during the last 20 years and future demographic developments predict more dramatic rises in the future. Due to the high economic burden of HF -especially in terms of inpatient costs- we need more compelling and innovative strategies to counteract the effects of HF. Reducing admission rates in acute HF is the most promising approach to decrease the economic burden of HF. In this context telemonitoring devices like a wireless pulmonary artery pressure monitoring system are promising new innovations [46–48]. As reported in a recently published study [49] this device has the potential to reduce the hospitalization rates for symptomatic HF by 37% and to lead to a significant cost reduction. The COI studies included in this review showed large variations in methodology used and the cost results vary as a consequence. Future COI studies would greatly benefit from a detailed presentation of cost components. Our review shows that there is a lack on cost data and further research is needed to highlight the economic burden of indirect costs of HF. High quality data from COI studies with a robust methodology applied can inform policy makers about the major cost drivers of HF and can be used as the basis of further economic evaluations.

## Additional files

Additional file 1: List of search terms, databases and results of each search. (DOCX 13 kb)

Additional file 2: Criteria used for inclusion/exclusion of reviewed articles. (DOCX 14 kb)

## Abbreviations

COI: Cost-of-Illness
HF: Heart failure
NYHA: New York Heart Association
