# Supplementary material for: Cost-of-illness studies in heart failure: a systematic review 2004–2016
Source: BMC Cardiovasc Disord. 2018 May 2;18:74. doi: 10.1186/s12872-018-0815-3 (PMC5930493; doi:10.1186/s12872-018-0815-3)
Supplement: Supplementary file 1 — List of search terms, databases and results of each search. (DOCX 13 kb) [file 12872_2018_815_MOESM1_ESM.docx]

| Search term / Database | PubMed  (Medline)  Search in „All Fields“ | Cochrane  Search in „Title, Abstract, Keywords“ | Science Direct  (Embase)  Search in „Title, Abstract, Keywords“ | Scopus  Search in „Title, Abstract, Keywords“ | CRD York  (incl. NHS EED)  Search in “Any Field” |
| --- | --- | --- | --- | --- | --- |
| „economics“ AND „heart failure“ | 1889 | 1 | 33 | 1269 | 286 |
| „cost of illness“ AND „heart failure“ | 274 | 11 | 29 | 568 | 7 |
| “cost” AND “heart failure” | 2612 | 22 | 1123 | 8156 | 416 |
| “economics” AND “cardiac failure” | 31 | 0 | 8 | 16 | 11 |
| “cost of illness” AND “cardiac failure” | 2 | 0 | 6 | 3 | 0 |
| “cost” AND “cardiac failure” | 62 | 1 | 349 | 131 | 13 |
| Total | 4870 | 35 | 1548 | 10143 | 733 |

List of search terms, databases and results of each search
