# Supplementary material for: Cost-of-illness studies in heart failure: a systematic review 2004–2016
Source: BMC Cardiovasc Disord. 2018 May 2;18:74. doi: 10.1186/s12872-018-0815-3 (PMC5930493; doi:10.1186/s12872-018-0815-3)
Supplement: Supplementary file 2 — Criteria used for inclusion/exclusion of reviewed articles. (DOCX 14 kb) [file 12872_2018_815_MOESM2_ESM.docx]

| Inclusion criteria | Exclusion criteria |
| --- | --- |
| 1. The study population is clearly described | 1. Studies with no cost data or a lack of costs referring to heart failure |
| 1. There is a well-defined research question, posed in an answerable form | 1. Cost-of-illness studies dealing generally with cardio-vascular diseases (CVD) |
| 1. The viewpoints of the analysis are clearly stated | 1. Reviews of existing economic studies related to heart failure |
| 1. There is enough economic detail in the study | 1. Letters / Commentaries on heart failure |
| 1. The chosen time horizon is appropriate for the inclusion of relevant costs | 1. Studies not reported in English or German |
| 1. The study is dealing with heart failure and its economic burden | 1. Cost-of-illness studies dealing only with systolic heart failure patients |
| 1. The main data sources are stated clearly | 1. Cost-effectiveness studies |
| 1. The costs are measured appropriately in monetary values |  |

Criteria used for inclusion/exclusion of reviewed articles
